# Diamond-like carbon (DLC) surface treatment decreases biofilm burden by *S. aureus* on titanium alloy in vitro*—* a pilot study

**DOI:** 10.1007/s00590-024-04093-4

**Published:** 2024-09-11

**Authors:** Anabelle Visperas, Kaixi Cui, Md. Masud Alam, Shonali Subramanian, Evan Butsch, Alison K. Klika, Anna Cristina Samia, Nicolas S. Piuzzi

**Affiliations:** 1 Cleveland Clinic Adult Reconstruction Research (CCARR), Department of Orthopaedic Surgery, Cleveland Clinic, 9500 Euclid Ave. A40, Cleveland, OH 44113 USA; 2 Department of Chemistry, Case Western Reserve University, 10900 Euclid Ave., Cleveland, OH 44106 USA

**Keywords:** Infection prevention, Infection, Biofilm, Implant coating

## Abstract

**Purpose:**

Periprosthetic joint infection is a complication of total joint arthroplasty with treatment costs over $1.6 billion dollars per year in the US with high failure rates. Therefore, generation of coatings that can prevent infection is paramount. Diamond-like carbon (DLC) is an ideal coating for implants as they are wear-resistant, corrosion-resistant, inert, and have a low friction coefficient. The purpose of this study was to test the efficacy of DLC surface treatment in prevention of biofilm on titanium discs infected with *Staphylococcus aureus *in vitro.

**Methods:**

Titanium alloy discs (*n* = 4 non-coated and *n* = 4 DLC-coated) were infected with 5 × 10^5^ colony-forming units (CFU) of *S. aureus* for 2 weeks then analysed via crystal violet and scanning electron microscopy (SEM).

**Results:**

Crystal violet analysis yielded differences in the appearance of biofilm on implant surface where DLC-coated had a clumpier appearance but no difference in biofilm quantification. Interestingly, this clumpy appearance did lead to differences in SEM biofilm coverage where significantly less biofilm coverage was found on DLC-coated discs (81.78% vs. 54.17%, *p* < 0.003).

**Conclusion:**

DLC-coated titanium alloy implants may have preventative properties in *S. aureus* infection. Observing differences in biofilm coverage does warrant additional testing including CFU titration and biofilm kinetics with eventual use in an animal model of periprosthetic joint infection.

## Introduction

Periprosthetic joint infection (PJI) is a complication of total joint arthroplasty (TJA) with high treatment cost, high failure rates, and reduced quality of life. PJI risk is highest during the early postoperative period but is a persistent risk throughout the lifetime of the joint ranging from 0.5 to 2.0% at 15 years [1]. With overall increases in the number of knee and hip replacements, the number of infections is expected to increase and cost over $1.85 billion annually by 2030 [2]. With current treatments including debridement, systemic antibiotics, local antibiotics, and staged revisions where implants are removed and replaced, failure rates are still quite high leading to over a 26% mortality rate at 5 years [1, 3].

The major culprit of failed treatment surgery is bacterial biofilm. This biofilm is produced by multiple bacterial species that are involved in PJI including gram-positive bacteria (82%)—*Staphylococcus aureus (S. aureus) and S. epidermidis,* coagulase-negative *Staphylococci* species, and gram-negative bacteria (11%)—*Enterobacterales* and *Pseudomonas aeruginosa* [1, 4]. Indeed, this biofilm growth on the implant surface makes bacteria over 1000 × less susceptible to antibiotics; therefore, staged revisions are the most successful treatment option for PJI [1, 5].

While overall infection rates have decreased with implementation of prophylactic strategies to limit infection, periprosthetic infections have not been eradicated. Therefore, alternative methods that can limit infection need to be developed that can provide long-acting protection outside the immediate surgical window, like antibacterial implant coatings.

Diamond-like carbon (DLC) is an ideal coating for implants as they are wear-resistant, corrosion-resistant, inert, and have a low friction coefficient and, therefore, has the potential to increase the life of the implant and benefit patients [6, 7]. These coatings can be introduced to a surface via two methods: plasma-based immersion ion implantation and deposition (PBIIID), which can coat a 3D surface or filtered cathodic vacuum arc (FCVA) which coats a planar surface [7]. These coatings have been tested for their wear-resistance, corrosive-resistance, biocompatibility, and inertness with various types of orthopaedic-related metals including cobalt chromium, titanium alloy, steel, and ultra-high molecular weight polyethylene (UHMWPE) [6–10].

These DLC coatings have intrinsic antibacterial properties by decreasing bacterial adhesion and can also be carriers for metal ions for increased antibacterial and antibiofilm effectiveness with implications in both primary and revision joint replacements [11]. DLC composites including silver [12], copper [12], silicone [13], titanium [14], etc., have also shown antibacterial effectiveness, and this effectiveness is not limited to metals as it can also be utilized on other materials such as polyurethane catheters [15], polyethylene [16], and textile silk bandages [17].

The purpose of this study is to test the efficacy of DLC surface treatment in prevention of biofilm on titanium discs infected with *Staphylococcus aureus (S. aureus) *in vitro.

## Methods

### Disc manufacturing

Discs were manufactured by Signature Orthopaedics. Discs were made of Grade 5 Ti-6Al-4 V ASTM B348. Discs were manufactured with solid carbide tooling using a Haas CNC machine and flat sanded on 600 grit aluminium oxide sandpaper. The disc dimensions were as follows: 12 mm major axis and 8 mm minor axis with 2.5 mm thickness. All finishes were machined finishes. Implants were gamma sterilized at Signature prior to shipment and use.

### Bacteria preparation

*Staphylococcus aureus* (ATCC 49525, ATCC, Manassas, VA) were cultured overnight in kanamycin sulphate (200 µg mL^−1^) Luria broth (LBK) with agitation at 200 rpm at 37 °C. Aliquots for experimentation were taken during the log-phase of growth based on optical density (OD).

### Biofilm culturing

Discs were placed into a 12-well dish with 5 × 10^5^ CFU *S. aureus* in 4 mL media. Samples were incubated at 37 °C with air supplement, without agitation. Two mL of media was replaced every other day without mixing the media prior to aspiration. For this pilot experiment, *n* = 4 non-coated discs and *n* = 4 DLC-coated discs were cultured for biofilm growth and separated for analysis into *n* = 2/group for crystal violet biofilm assay quantification and *n* = 2/group for scanning electron microscopy (SEM) imaging analysis.

### Crystal violet assay

Media was aspirated from each well and gently washed using PBS to remove any non-adherent planktonic bacteria. Discs were submerged in 1 mL of 5% crystal violet solution for 20 min then aspirated and rinsed. Discs were left to dry and photographed for qualitative analysis. To quantify biofilm, discs were submerged in 1 mL of 30% acetic acid to solubilize the crystal violet for 15 min, diluted 8 × with deionized water, and OD was measured at 595 nm.

### Scanning electron microscopy

SEM processing, imaging, and analysis were completed based on standardized methods done previously [18]. In short, discs were fixed with 4% paraformaldehyde and dehydrated using an ethanol soaking series. Samples were dried and sputter coated with 25 nm of gold and analysed using a Zeiss SIGMA VP-FESEM (White Plains, NY) using a custom script written in DigitalMicrograph software (Gatan Inc., Pleasanton, CA) to automate the SEM stage and image capture. Twenty images were collected at 1500 × magnification and 5 kV from the top surface of the disc.

Each SEM image was segmented using the Trainable Weka Segmentation plugin in Fiji (distribution of ImageJ, NIH, Bethesda, MD). Classifier was previously trained on 25 images to distinguish biofilm-present and biofilm-absent sections. The segmentation result was generated and analysed by the per cent area coverage calculator on Fiji [18].

### Statistics

Not all groups were normally distributed (based on Shapiro–Wilk test). Therefore, nonparametric Kruskal–Wallis ANOVA with Dunn’s post-test was done using GraphPad Prism 8.0 (San Diego, CA).

## Results

**Fig. 1:**
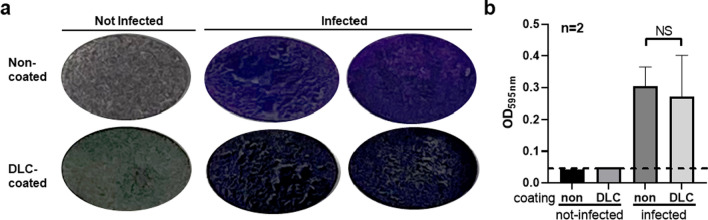
Crystal violet biofilm analysis yields no difference in biofilm. Discs were incubated with 5 × 10^5^ CFU *S. aureus* for 2 weeks. (**A**) Discs were washed with saline and stained with crystal violet for biofilm visualization. (**B**) Discs were subsequently soaked in acetic acid for crystal violet quantification. NS = not significant

Qualitative assessment of biofilm via crystal violet showed a less uniform, clumpier biofilm coverage on DLC-coated discs compared to non-coated discs (Fig. 1A). Of note, DLC-coated discs were darker in colour compared to non-coated discs. Quantitative assessment of biofilm via optical density showed no difference in OD between groups (non-coated OD = 0.304 and DLC-coated OD = 0.272; Fig. 1B).

**Fig. 2:**
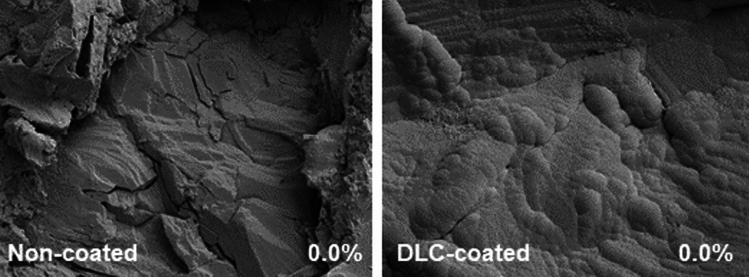
SEM imaging of non-infected discs for visual analysis representative SEM imaging at 1500 × magnification of non-infected discs

**Fig. 3:**
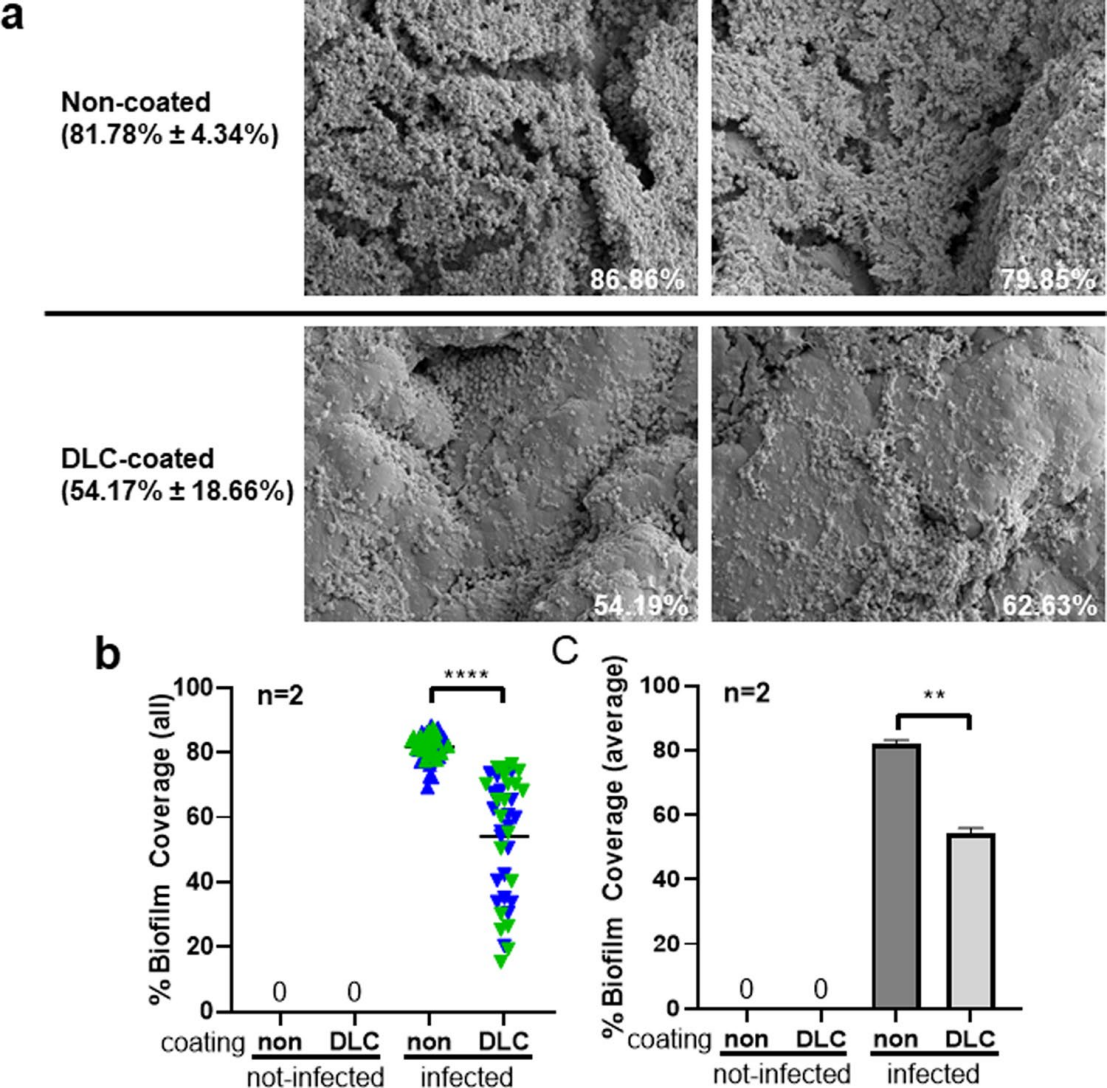
SEM biofilm analysis yields significant difference biofilm coverage. Discs cultured with *S. aureus* for 2 weeks were washed after incubation, fixed, and dehydrated. Samples were gold sputtered, and 20 images were taken at specified locations on the top of the disc (accounting for ~ 0.5% of the total area of the disc). **A** Representative images taken at 1500 × magnification. Per cent depicted is mean ± standard deviation. **B** Per cent coverage of each individual image. All green symbols were results from images acquired from disc #1, and the blue symbols were images acquired from disc #2 from each group. **C** Average biofilm coverage of all images taken per disc. ****p < 0.0001 and **p < 0.01

Baseline SEM imaging on non-infected discs showed that DLC coating did modify the surface of the discs where surface was smoother, and crevices were not as apparent compared to non-coated (Fig. 2). Upon infection, qualitative assessment did show differences in appearance of the biofilm between groups where biofilm on DLC-coated discs did not look as densely packed (Fig. 3A). Indeed, when imaged and quantified, areas of biofilm coverage were significantly decreased in the DLC-coated discs compared to the non-coated discs when all images were analysed separately (non-coated biofilm coverage = 81.78% ± 4.34% vs. DLC-coated biofilm coverage = 54.17% ± 18.66%; *p* < 0.0001; Fig. 3B) or averaged per disc (non-coated biofilm coverage = 81.78% ± 1.32% vs. DLC-coated biofilm coverage = 54.17% ± 1.690%; *p* = 0.0030; Fig. 3C).

## Discussion

With the increasing volumes of joint replacement surgeries globally, accompanied by persistent gaps in infection prevention strategies and the need for more effective treatments, addressing PJI has become an urgent priority in orthopaedic practice [19]. With high failure rates during PJI treatment, novel prophylactic treatments like antibacterial implant coatings are needed to decrease the risk of infection outside the perioperative window [20]. In this study, we have shown in vitro that DLC surface modification on titanium alloy can significantly decrease *S. aureus* biofilm coverage after 2 weeks of culture.

Applications for DLC coatings are appealing due to their mechanical, bio-inert, and antibacterial properties. DLC’s anti-wear and non-corrosive characteristics have the potential to lengthen the life of implants and decrease cellular reactivity to wear particles. Their use has been shown to be biologically inert, non-toxic towards osteoclasts, and promote bone mineralization in vitro providing rationale of its usefulness clinically without negative biological side effects. Of note, the previous reports on wear resistance with DLC and UHMWPE used an old formulation of UHMWPE where newer formulations have optimized its shortcoming and, therefore, do require additional testing with the new formulation to assess its wear resistance in combination [7].

Various ions can be supplemented during DLC coating manufacturing to increase antibacterial efficacy [11]. Both silver and copper nanoparticle-impregnated DLC leads to rapid release of silver and copper ions, respectively, leading to a reduction of both surface-bound and planktonic bacterial species in vitro via metabolism disturbance and membrane destabilization mechanisms [12, 21]. Fluorine-DLC and silicone-DLC coating creates a hydrophobic surface thus reduces the surface free energy and bacterial adhesion [11]. Zinc oxide nanoparticles embedded into DLC antibacterial coatings produce an adaptive release of Zn^2+^ ions when in an aqueous environment leading to acidosis and toxicity to *Staphylococci* species [22]. Titanium dioxide-doped DLC has bactericidal effects via oxidative damage to the cell wall and decreases the interfacial energy of bacterial adhesion in a dose-dependent manner [23].

Indeed, other implant coatings are also under investigation. Antifouling or antiadhesion coatings are being developed that focus on the surface hydrophilic/hydrophobic properties, conductivity, and surface energy. Polymers such as polyethylene glycol (PEG), zwitterionic polymers, hyaluronic acid (HA), and sodium alginate increase hydrophilicity and, therefore, decrease the binding sites for bacteria [11]. Zwitterionic polymers contain isoelectric characteristics that repel charged proteins and bacteria, and their quaternary ammonium salts such as phosphorous, pyridine, and imidazole also have antibacterial properties. Polysaccharides such as HA and sodium alginate inhibit bacterial adhesion through electrostatic repulsion interactions [11]. Topographic 3D nanostructures such as blunt nanopillars, spikes, and nanoedges have shown success where bacteria adhesion is disrupted, causing stress and rupture of the cell membrane [24]. Of note, this may not work on all bacteria with thicker cell membranes. Recently, a point-of-care antibiotic-loaded antimicrobial coating that is applied during surgery that couples PEG with poly(allyl mercaptan) (PEG-PAM) polymers prevented in vivo infection in both mouse models of arthroplasty and spine surgery without inhibiting osseointegration [25]. Hydrophobic coatings like fluorinated polymers produce a low surface energy coating that reduces interactions between bacteria and the surface [26]. Use of metal oxides such as TiO_2_, CuO, AgO, and ZnO that release reactive oxygen species upon irradiation and metal ion release can inhibit bacterial adhesion without affecting osteogenesis [14, 27]. Indeed, coatings using of immobilized silver ions on hydroxyapatite film on polymers have also shown bactericidal and anti-biofilm effects without high-temperature processing for coating application [28]. Of note, majority of the DLC composite literature has focused on antibacterial effects associated with metal ion mechanisms and have not explored composites with alternative bactericidal agents.

Addition of bactericidal agents to implant coatings is also another area of active research. In this case, adhered bacteria are destroyed via an active substance covalently linked or adsorbed in the coating including chitosan, antimicrobial peptides (AMPs), or quaternary ammonium compounds (QACs) [11]. A majority of AMPs and QACs are cationic and can rapidly penetrate the negatively charged bacterial cell membrane causing autolysis and cell death, efficiently killing bacteria regardless of antibiotic susceptibility with low toxicity [29–31]. Chitosan is a cationic polysaccharide where its positively charged amino groups generate electrostatic interactions with bacteria thus altering permeability of the cell wall and lysis [32]. Indeed, while this method directly kills bacteria, accumulation of dead bacteria and their intracellular products may potentially limit these coatings long term by forming a barrier between the surface and subsequent bacteria thus giving bacteria an opportunity to adhere and create a biofilm.

Controlled-release antibacterial agents in coatings have the potential to be used over an extended period post-implantation. These coatings can be carriers of antibacterial agent which can be released through diffusion or degradation including antibiotics, metal ions, fluorine, and iodine^24^. Additionally, these nanomaterials can be responsive to external stimuli including magnetic fields, light, or temperature creating a temporal effect when needed. Hydrogels, hydrophilic 3D polymeric structures, and polyelectrolyte multilayers (PEMs) are made with biologically relevant materials such as chitosan, collagen, and hyaluronic acid and can be loaded with various antibacterial agents that can be used as coatings on implant surfaces with sustained release of its load but still needs further characterization [31]. Nevertheless, additional characterization of the coating process needs to be investigated to ensure that the implant’s mechanical and osseointegrative integrity is still maintained.

This study is not without limitations. This pilot study had a small sample size of *n* = 2 per group and was completed in a single experiment. This study will need to be repeated to confirm reproducibility. This study only had a single timepoint for biofilm readout. Whether attachment and kinetics of biofilm growth is altered at earlier timepoints needs to be addressed. This study also used a higher CFU than would be required for infection seeding in clinical settings. Nevertheless, results did show a difference in biofilm coverage at this high CFU, suggesting that larger differences may be apparent with smaller inoculum. Additionally, these experiments were completed in static conditions and whether different results will be obtained under constant flow conditions needs to be investigated.

## Conclusion

Although many studies have been published on DLC coating of metal implant materials in the context of joint arthroplasty, this is the first evidence showing that DLC surface treatment significantly decreases *S. aureus* biofilm coverage on titanium discs after 2 weeks of culture via systematic SEM analysis. Although its effect was not readily apparent via crystal violet analysis, its growth differences and seeding on the surface of the implant were apparent with SEM analysis. These data have warranted further investigation into the usefulness of DLC coating in orthopaedic implants to prevent PJI.
